# Prevalence and risk factors of transmission of hepatitis delta virus in pregnant women in the Center Region of Cameroon

**DOI:** 10.1371/journal.pone.0287491

**Published:** 2024-06-20

**Authors:** Juliette-Laure Ndzie Ondigui, Nadège Mafopa Goumkwa, Cindy Lobe, Brigitte Wandji, Patrick Awoumou, Prisca Voussou Djivida, Puinta Peyonga, Solange Manju Atah, Vivian Verbe, Rachel Kamgaing Simo, Sylvie Agnès Moudourou, Ana Gutierrez, Rosi Garcia, Isabelle Fernandez, Sara Honorine Riwom Essama, Robinson Mbu, Judith Torimiro

**Affiliations:** 1 Faculty of Sciences, Department of Microbiology, University of Yaoundé 1, Yaounde, Cameroon; 2 Chantal BIYA” International Reference Center for Research on HIV/AIDS Prevention and Management (CIRCB), Yaounde, Cameroon; 3 Department of Food Science and Nutrition, National School of Agro-Industrial Sciences, University of Ngaoundere, Ngaoundere, Cameroon; 4 Yaoundé Gyanecology Obstetrics and Paediatrics Hospital, Yaounde, Cameroon; 5 Faculty of Medicine and Biomedical Sciences, Department of Biochemistry, University of Yaounde 1, Yaounde, Cameroon; 6 Faculty of Medicine and Biomedical Sciences, Department of Public Health, University of Yaounde 1, Yaounde, Cameroon; 7 Cité Verte District Hospital, Yaounde, Cameroon; 8 Bikop Catholic Health Center, Bikop, Cameroon; 9 Faculty of Medicine and Biomedical Sciences, Department of Gynecology/Obstetrics, University of Yaounde 1, Yaounde, Cameroon; Centre de Recherche en Cancerologie de Lyon, FRANCE

## Abstract

**Background:**

Hepatitis B virus (HBV) and hepatitis delta virus (HDV) co-infection has been described as the most severe form of viral hepatitis, and can be co-transmitted from mother-to-child. A seroprevalence of 4.0% of HDV infection was reported in pregnant women in Yaoundé, and 11.9% in the general population in Cameroon. Our objective was to describe the rate of HDV infection in HBsAg-positive pregnant women and to determine risk factors associated with mother-to-child transmission of HDV.

**Materials and methods:**

A cross-sectional, descriptive study was conducted from January 2019 to July 2022 among pregnant women attending antenatal contacts in seven health structures in the Centre Region of Cameroon. A consecutive sampling (non-probability sampling) was used to select only pregnant women of age over 21 years, who gave a written informed consent. Following an informed consent, an open-ended questionnaire was used for a Knowledge, Attitude and Practice (KAP) survey of these women, and their blood specimens collected and screened for HBsAg, anti-HIV and anti-HCV antibodies by rapid tests and ELISA. HBsAg-positive samples were further screened for HBeAg, anti-HDV, anti-HBs, and anti HBc antibodies by ELISA, and plasma HDV RNA load measured by RT-qPCR.

**Results:**

Of 1992 pregnant women, a rate of 6.7% of HBsAg (133/1992) with highest rate in the rural areas, and 3.9% of hepatitis vaccination rate were recorded. Of 130, 42 (32.3%) were anti-HDV antibody-positive, and 47.6% had detectable HDV RNA viraemia. Of 44 anti-HDV-positive cases, 2 (4.5%) were co-infected with HBV and HCV, while 5 (11.4%) with HIV and HBV. Multiple pregnancies, the presence of tattoos and/or scarifications were significantly associated with the presence of anti-HDV antibodies. Of note, 80% of women with negative HBeAg and positive anti-HBe serological profile, had plasma HDV RNA load of more than log 3.25 (>10.000 copies/ml).

**Conclusion:**

These results show an intermediate rate of HDV infection among pregnant women with high level of HDV RNA viremia, which suggest an increased risk of vertical and horizontal co-transmission of HDV.

## Introduction

Hepatitis B virus (HBV) is the aetiologic agent of hepatitis B. The WHO estimates that 296 million people were living with chronic hepatitis B in 2019 with 1.5 million new infections each year [1], whereas Hepatitis delta virus (HDV) affects nearly 5% of people with chronic hepatitis B worldwide [1]. HDV requires the presence of HBV in order to replicate [2–4] in co-infection or super-infection causing the most serious form of chronic viral hepatitis, given its more rapid progression to hepatocellular carcinoma and death [5–8]. It is also worth noting, the low HBV vaccination rate among pregnant women in Cameroon of less than 2.5% [9].

HBV and HDV can be transmitted through skin lesions (injection, scarification, tattooing), contact with infected blood or blood products, but can also be co-transmitted through sexual intercourse and from mother-to-child [10]. Mother-to-child transmission of HDV is rare but may increase with high level of plasma load of HBV DNA and HDV RNA in the pregnant woman, and in individuals with multiple sexual partners and/or with scarification [11]. Therefore, pregnant women are an index population for both vertical and horizontal transmission of HBV and HDV. However, certain practices favour the transmission of HBV in the Centre Region of Cameroon. They include poor accessibility to healthcare, low rate of vaccination [9], low rate of condom use, age of first pregnancy and early first sexual intercourse, high rate of home delivery, poor link to prevention of mother-to-child transmission (PMTCT) activities and to other maternal-infant disease prevention efforts in Cameroon [20].

Vaccination against hepatitis B is an effective strategy to prevent HDV infection. Therefore, scaling-up the national vaccination Programme against hepatitis B in the general population, could lead to a drop in the incidence of hepatitis D worldwide. In 2018, it was reported that 83.3% of pregnant women attending ANC in Yaoundé, Cameroon, knew that vaccination against HBV infection is a method of prevention, and 47.1% knew that HBV could be transmitted from mother-to-child[12]. In Cameroon the seroprevalence of HBV (HBsAg) in the general population is 11.2%, about three times higher than that of the human immunodeficiency virus (HIV) of 3.4% [13,14]. In 2021, the Cameroon Ministry of Public Health reported a seroprevalence of HBsAg among pregnant women at first trimester of 5.0% in the Centre Region where this study was carried out against 8.63% of national prevalence [12]. In addition, in Central Africa, HDV seroprevalence is estimated at 25.64% in the general population [7], compared to 46.73% in Cameroon in 2018 [15].

Therefore, low hepatitis B vaccination coverage and lack of recent epidemiological data on HDV in pregnant women, show the need to strengthen ongoing prevention strategies to mitigate risk of vertical and horizontal transmission of HBV and HDV. Thus, our specific objectives were to determine the rates of HDV infection and factors associated with risk of HDV infection among HBsAg positive pregnant women.

## Materials and methods

### Study design, enrollment procedure and eligibility criteria

A cross-sectional study was conducted from January 2019 to July 2022 in a tertiary hospital, five District hospitals, and one rural Health Center in the Center Region of Cameroon, among pregnant women attending ANC. A total of 1992 pregnant women were enrolled from an anticipated 1475 participants deduced from a statistical formula shown below [16] which is commonly used to determine the minimum sample size in medical studies:

n = (Zα)2 p (1- p)/d2 where:

Zα = standard normal variate (1.96 for a 95% confidence interval);

p = prevalence of HDV among pregnant women in Cameroon (4.0) [9];

d = precision of the estimate (0.01).

Before enrolling the participants, a group sensitization at the hospital or Health Center was conducted to raise awareness on hepatitis B transmission and prevention, hepatitis B and pregnancy, the importance of HBV screening and the purpose of the study. Thereafter, a Consent Form and a Questionnaire with open-ended questions were administered to collect personal and socio-demographic data, their knowledge, attitude and practices with respect to hepatitis B. Some women who took part in the sensitization campaign were not enrolled in the study because they had to have the opinion of their partners, or did not give any reason.

A consecutive sampling (non-probability sampling) was used to select the pregnant women. In order to avoid any confusion and to preserve the anonymity of the participants. Each participant was assigned a code that allowed us to link each questionnaire to its corresponding blood specimen, while the Register of the ANC used only by hospital staff was established to link the subjects to their results. This same code was used throughout the analysis in the laboratory and the results were linked to the identity of the subject by the hospital staff and not by the research team.

### Laboratory procedures

After completing the Questionnaire, 5 ml of blood was drawn from 1992 subjects into tubes containing ethylene-diamine-tetra-acetic acid (EDTA), processed and the plasma tested for HBsAg, anti-HIV and anti-HCV antibodies (because these viruses are blood-borne and can favour co-infection and even co-transmission with HDV [17]) using rapid diagnostic tests (RDT, ABON, Determine HIV1/2 and KHB Diagnostic kit for HIV antibodies 1 + 2) and ELISA (Murex HBsAg, and Murex HCV Ab), following the manufacturer’s instructions. Furthermore, the HBsAg-positive samples were tested by ELISA for other HBV infection biomarkers: anti-HBc, HBeAg, and anti-HBe using the appropriate kits by Dia.Pro Diagnostic Bioprobes srl, Sesto San Giovanni, Italy. Positive and negative plasma controls were run alongside each test, following the National Testing Algorithms for these viruses in Cameroon.

ELISA was performed to detect anti-HDV antibodies in 130 plasma samples (HBsAg-positive) using the ETI-DELTA-IGMK-2 kit (Dia.Pro Diagnostic Bioprobes srl, Sesto San Giovanni, Italy). Quantification of HDV RNA (viral RNA load) by real-time qRT-PCR (Eurobioplex HDV Kit, Eurobio Scientific, Les Ulis, France) was performed using 34 anti-HDV positive plasma samples. All results from serologic testing were made available to the women within two weeks after specimen collection. The rapid test results were confirmed by two technicians and the samples with borderline results, were further analyzed by ELISA. Plasma HDV RNA load was quantified by qRT-PCR commercial kit that detects all 8 main genotypes of HDV, with a lower limit of detection of 100IU/mL.

All women who tested positive for HBsAg were offered post-test counselling on their status, modes of transmission of hepatitis B, need for testing of close contacts for hepatitis B, and vaccination of baby within the first twenty four hours of life against hepatitis B. They were then referred for clinical evaluation and management by a gastroenterologist, and those who were negative for HBsAg were advised to get vaccinated.

### Statistical analysis and interpretation of results

Data collected was regularly checked, corrected, recorded and processed using IBM.SPSS. Statistics.v20 software, while ensuring anonymity with the use of subject registration code. Frequencies of qualitative data were estimated as means. Using internal negative and positive controls in each run, a cycle threshold (Ct) of ≤ 35 was considered positive, and Ct > 35 and ≤ 45 was considered a non-quantifiable positive result. Using a standard curve drawn from standards provided by the manufacturer, the HDV RNA load was extrapolated to copies/mL which was then multiplied by a factor (0.18) to convert into IU/mL. The analysis of the relationship between the prevalence and the different characteristics of the sample was done by logistic regression. Using the Pearson Chi-2 test, the factors associated with HBV and HDV infection, were determined with p values <0.05 considered statistically significant. Ten samples with missing data were not included in the analysis.

### Ethical considerations

The study was conducted in accordance with the fundamental principles of the Declaration of Helsinki[18], with administrative and ethical approval from health facilities and laboratories, as well as the National Ethics Committee for Human Health Research in Cameroon (N° 2018/06/1150/L/CNERSH/SP), respectively. A written informed consent was obtained from all participants, data was recorded using unique identifiers to ensure confidentiality, and laboratory results were returned to participants for potential benefits in their clinical management. The subjects who were positive for HBsAg antigen were referred to a gastroenterologist and those HBsAg-negative and not vaccinated were advised to get vaccinated.

## Results

### Socio-demographic parameters and knowledge, attitude and practices of pregnant women

Overall, 1992 pregnant women were tested for HBsAg, with a mean age of 27.53 ± 5.68 years. The average gestational age was 17 ± 6 weeks, with a majority of the women at the second trimester pregnancy. One thousand nine hundred and thirteen (1913) women have never received the hepatitis B vaccine, but 56 had received three doses of the vaccine (2.8%) (Table 1). One thousand six hundred and twenty seven (1627) women were screened for the first time for HBsAg during this study (81.7%). In the group of HBsAg-positive women, 53(39.9%) women were in their first pregnancy, 75 (56.4%) lived with a partner, 37 (27.8%) knew their partner’s hepatitis B status and 5.4% (2/37) of their partners were positive for HBsAg, 15 (11.3%) had already been tattooed or scarified and 21 (15.7%) had a history of surgery.

**Table 1:** Characteristics of the study population.

Variables	HBsAg					
			Unadjusted		Adjusted	
	Negative (%)	Positive (%)	OR (95% CI)	P–value	aOR (95% CI)	P–value
**Age (years)**						
15–20	209 (91.2)	19 (8.8)	1			
21–25	580 (92.2)	49 (7.8)	0.87 (0.54–1,38)	0.547		
26–30	507 (94.9)	31 (5,6)	0.613 (0.37–1,01)	0.054		
31–35	360 (93.0)	27 (7.0)	0.726 (0.43–1,23)	0.232		
36–40	172 (94.0)	11 (6.0)	0.671(0.34–1,33)	0.252		
41–45	31 (100.0)	0 (0)	/	0.998		
**Gestational age (weeks)**						
1–14	527(92.1)	45(7.9)	1		1	
15–28	940(94.4)	65(5.6)	0.96 (0.65–1.41)	0.833	0.84 (0.54–5.80)	0.43
29–42	374(90.1)	41(9.9)	2.10 (1.2–2.92)	**0.006** *	1.631(1.02–2,62)	**0.04** *
**Hepatitis B vaccination**						
Not vaccinated	1779(93.04)	133(7.0)	1			
1 Dose	10(100,0)	0(0)	/	0.999		
2 Doses	14(100,0)	0(0)	/	0.999		
3 Doses	56(100,0%)	0(0)	0.24 (0.03–1.18)	0.16		
**Location of health institution/study site.**						
urban	1169(94.88)	63(5,1)	1			
semi-urban	263(91.96)	23(8,0)	1.63 (0.99–2.67)	0.06	2.23 (1.3–3.8)	**0.003** *
rural	427(90.08)	47(9.9)	1.97 (1.38–2,85)	**0.001** *	2.09 (1.42–3.08)	**0.001** *
**First time of HBsAg screening**	1557(95.7)	70(4.3)				

-*.*The Chi-square statistic is significant at the* .*05 level*.

### Prevalence of HDV and HBV infection markers

Of 1992 pregnant women tested for HBsAg, 133 were positive (6.7%) while 9.8% of women in the third trimester were HBsAg-positive, and 9.9% of them live in the rural area. Of these, 42 samples were positive for anti‐HDV antibodies by serology, and 27 (62.5%) had detectable plasma HDV RNA. Meanwhile, rates of 9.0%, 79.7% and 94.0% for HBeAg, anti-HBe and anti-HBc, respectively, were recorded for the HBsAg-positive specimens. Table 2 shows the distribution of serological markers of hepatitis B infection as a function of the presence of anti-HDV antibodies, with no statistically significant association of HBeAg and anti-HDV presence (p = 0.169). Two (4.8%) women were co-infected with HCV, HBV and HDV, and 5 (11.9%) with HIV, HBV and HDV were identified. However, HBV/HIV and HBV/HCV co-infections were not associated with anti-HDV positivity in this study. In univariate analysis, neither HIV nor HCV serologic positivity was associated statistically significantly with the presence of anti-HDV antibodies (Table 2).

**Table 2:** Distribution of viral infections (HCV, HDV, and HIV) and serological markers of hepatitis B infection among HBsAg-positive pregnant women.

Serological markers		Presence of anti-HDV antibodies		
		Negative (%)	Positive (%)	P-value
**anti-HIV**	Negative	80(68.4)	37(31.6)	0.756
	Positive	8(61.5)	5(38.46)	
**anti-HCV**	Negative	88(68.8)	40(31.2)	0.103
	Positive	0(0)	2(100.0)	
**HBeAg**	Negative	82(69.5)	36(30.5)	0.169
	Positive	6(50.0)	6(50.0)	
**anti-HBc**	Negative	2(25.0)	6(75.0)	**0. 014** ^*^
	Positive	86(70.5)	36(29.5)	
**anti-HBe**	Negative	9(36.0)	16(64.0)	**0.001** ^*^
	Positive	79(75.2)	26(24.8)	
**HBeAg-/anti-HBe -**		8(44.4)	10(55.6)	**< 0.001** *
**HBeAg+/anti-HBe -**		0(0)	6(100.0)	
**HBeAg-/anti-HBe +**		75(75.3)	26(25.7)	
**HBeAg+/anti-HBe +**		5(100.0)	0(0)	

*Note* **+**: positive; **-**: negative

*.The Chi-square statistic is significant at the .05 level.

The Pearson’s Chi-square test was used for covariates with frequencies >5, while covariates with frequencies ≤ 5, the Fisher’s Exact test analysis was performed.

### Factors associated with hepatitis delta virus infection

Our study shows that 4 women with more than 5 children were positive for anti-HDV antibodies. In addition, women living with a partner (44.0%) were had a higher risk of being exposed to HBV and HDV than women living alone (16.4%; p = 0.001), while women who were not tattooed or scarred had a 0.26 less chance of having anti-HDV (p = 0.02), just as women who had not undergone surgery had a 0.18 less chance of having anti-HDV antibodies (p = 0.027). (Table 3). Comparing the seroprevalence of HDV antibodies against biomarkers of HBV infection in the HDV antibody-positive versus HDV antibody-negative group, there was a statistically significant difference with the presence of anti-HBc (p = 0.014) and anti-HBe (p = 0.001). Of note, the HBeAg-negative/anti-HBe-positive profile was the most frequently detected amongst those with plasma HDV RNA viral load of more than log 3.25 (10.000 copies/mL) (Table 4).

**Table 3:** Variation of prevalence of hepatitis delta according to the clinical characteristics of pregnant women (N = 130).

Variables	Presence of anti-HDV					
			Unadjusted		Adjusted	
	Negative (%)	Positive (%)	OR (95% CI)	P–value	aOR (95% CI)	P–value
**Age (years)**						
15–20	16(84.2)	3 (15.8)	1			
21–25	31 (66.0)	16 (34.0)	2.462 (0.80–7.78)	0.116		
26–30	17 (60.7)	11 (39.3)	2.696 (0.84–8.69)	0.097		
31–35	18 (69.2)	8 (30.8)	1.852 (0.55–6.27)	0.322		
36–40	6 (60.0)	4 (40.0)	2.778 (0.591–13.05)	0.192		
**Gestational age (weeks)**						
1–14	18 (69.2)	8 (30.2)	1			
15–28	34 (68.0)	16 (32.0)	1.179 (0.45–3.05)	0.734		
29–42	36 (66.7)	18 (33.3)	1.474 (0.54–4.0)	0.446		
**Number of life births**						
0	40 (75.5)	13 (24.5)				
1	40 (75.5)	10 (27.8)	1	0.999		
2	10 (62.5)	6 (37.5)	/	0.999		
3	11 (55.0)	9 (45.0)	/	0.999		
4	1 (100.0)	0 (0.0)	/	0.999		
5+	0 (0.0)	4 (100.0)	/	0.999		
**Marital status**						
Alone	46 (83.6)	9 (16.4)	1		1	
married	42 (56.0)	33 (44.0)	4.016 (1.72–9.37)	**0.001** *	5.414 (2.15–13.65)	0.0001*
**Tattoo**						
Yes	6 (40.0)	9 (60.0)	1			
no	82 (71.3)	33 (28.7)	0.268 (0.088–0.813)	**0.02** *	0.22 (0.06–0.76)	**0.016** *
**History of surgical operation**						
Yes	19 (90.5)	2 (9.5)	1			
No	69 (63.3)	40 (36.7)	0.182 (0.04–0.082)	**0.027** *	7.03 (1.45–34.06)	**0.015** *
**Cicumcision**						
Yes	5 (100.0)	0 (00.0)	1			
no	83 (66.4)	42 (33.6)	/	0.999		
**Consumption of alcohol**						
yes	39 (68.4)	18 (31.6)	1			
no	49 (67.1)	24 (32.9)	1.061 (0.51–2.23)	0.875		
**Aware of hepatitis B status of close people**						
no one knows	2 (100.0)	0 (00.0)	1			
husband	15 (60.0)	10 (40.0)	/	0.999		
family member	0 (00.0)	2 (100.0)	/	0.999		
friend	46 (64.8)	25 (35.2)	/	0.999		

Note.

*.*The Chi-square statistic is significant at the* .*05 level*.

**Table 4:** Plasma HDV RNA viral load as a function of the serological profile of anti-HBe and HBeAg markers.

Serological profile	HDV RNA viral load (copies/mL)					*P value*
	Not detected	< 15	(15–1000)	(1000–10000)	(10000–35000)	
**HBeAg-/anti-HBe -**	5(33.33%)	0(0.0%)	2(40.0%)	2(40.0%)	0(0.0%)	0.502
**HBeAg+/anti-HBe -**	1(6.67%)	0(0.0%)	1(20.0%)	0(0.0%)	1(20.0%)	
**HBeAg-/anti-HBe +**	9(60.0%)	4(100.0%)	2(40.0%)	3(60.0%)	4(80.0%)	
**Total**	15	4	5	5	5	

*p value* (Fisher’s exact test).

## Discussion

HDV infection in pregnancy can lead to a higher risk of preterm birth, increased risk of perinatal transmission, and increased risk of liver complications for the mother and baby [19]. We therefore sought to determine the occurrence of HDV infection and associated risk factors among some pregnant women in the Centre Region of Cameroon. The overall 6.7% seroprevalence of HBsAg observed in this study is similar to that obtained by Torimiro et al in 2018 (6.4%) among pregnant women in Yaoundé, Cameroon [9]. In comparison to other African countries with similar socio-economic and public health contexts, such as Nigeria, Gabon and Burkina Faso, similar prevalence has been reported [20,21]. We also noted that in our study population the seroprevalence of HBsAg is higher among pregnant women in rural areas compared to those in urban areas. Other studies conducted in rural areas in Cameroon, have recorded higher prevalence of HBsAg ranging from 7.5% to 13.7% [22–24].

This moderate to high prevalence of hepatitis B in rural areas in Cameroon could be related to the low level of knowledge about hepatitis B modes of transmission and prevention, and risk factors of attitude and practices including idleness and promiscuity. In other words, these are factors that would favour transmission of HDV [25]. Although the efficacy of the hepatitis B vaccine has been proven and has been available since 2005 in the Cameroon EPI [26], this study shows that vaccination coverage remains very low among women of child-bearing age, which contributes in keeping the pool of index infected sub population on the increase. It is therefore not surprising to detect the high prevalence of HDV infection in pregnant women in the Centre Region of Cameroon. Since 1991 when the first study on the prevalence of HDV was conducted in Cameroon [27], several studies have been carried out in the general population or among people with particular socio-demographic characteristics such as pygmies. The variations in the prevalence of anti-HDV antibody ranged from 10.5% to 69% in Cameroon [11,15,28–30]. On the other hand, very few of these studies have focused on pregnant women who are an index for both horizontal and vertical transmission of HBV and HDV. These studies reported low levels of anti-HDV antibodies in pregnant women of 2.3–4.0% [9,27]. These values are low compared to the rate of 32.3% anti-HDV antibody prevalence which we obtained in our study. This difference could be due to the fact that the previous studies were conducted in the Yaoundé an urban area, where there are better living conditions, higher level of education, awareness and knowledge of hepatitis B compared to the semi-urban and rural areas that were included in our study. In comparison of anti-HDV antibody rates among pregnant women in other African countries such as Benin (11.4%), Mauritania (14.7%), Gabon (15.6%), the Central African Republic (21. 4%)[31–34], our result is higher at 32.3%.

Our findings show that the number of pregnancies, marital status, presence of tattoos or scarifications and history of surgery are factors associated with the positivity of anti-HDV antibodies. Besombes and colleagues reported in 2020 that individuals with more than 10 sexual casual partners and those who received injections were most at risk of getting HDV [11]. `HIV and HCV co-morbidities have been shown to increase the chance for HDV acquisition [17]. However, these risk factors (presence of HIV and HCV) were assessed but no association was observed in this study.

The rates of HBV serological markers in anti-HDV-positive individuals showed that anti-HBc antibodies and anti-HBe antibodies were statistically associated with anti-HDV positivity in this study. Similarly, a majority of women with the HBeAg-negative/anti-HBe-positive serological profile, had plasma HDV RNA viral load ranging from 10000 to 35000 copies/mL.

The unavailability of data on HDV genotypes constitutes the main limitation of this study.

## Conclusion

In conclusion, the results of this study show that in spite of the availability of the hepatitis B vaccine in the EPI in Cameroon, HBV infection is moderately endemic (6.7%) in the Centre Region of Cameroon, while HDV rate is 32.3% among pregnant women. Hence, the need to intensify sensitization and control strategies for hepatitis B and hepatitis delta among pregnant women and contribute to the global elimination of hepatitis B.

## Supporting information

S1 File (DOCX)
