# Supplementary material for: Prevalence and risk factors of transmission of hepatitis delta virus in pregnant women in the Center Region of Cameroon
Source: PLoS One. 2024 Jun 20;19(6):e0287491. doi: 10.1371/journal.pone.0287491 (PMC11189217; doi:10.1371/journal.pone.0287491)
Supplement: S1 File — (DOCX) [file pone.0287491.s001.docx]

**Data on Hepatitis delta virus in pregnant women - PONE-D-23-15170**

| **Code** | **Date of sample collection** | **HDV antibody serology** | **HBsAg serology** | **HDV viral load (log)** | **HDV viral load (copies/mL)** |
| --- | --- | --- | --- | --- | --- |
| PTMEVHB706 | 07/08/2020 | Positif | Positif | Non Detectable | Non Detectable |
| PTMEVHB801 | 27/08/2008 | Positif | Positif | Non Detectable | Non Detectable |
| PTMEVHB793 | 26/08/2020 | Positif | Positif | Non Detectable | Non Detectable |
| PTMEVHB183 | 29/04/2019 | Positif | Positif | Non Detectable | Non Detectable |
| MFO035 | 29/07/2019 | Positif | Positif | Non Detectable | Non Detectable |
| MFO021 | 25/07/2019 | Positif | Positif | Non Detectable | Non Detectable |
| MBY038 | 29/07/2019 | Positif | Positif | Non Detectable | Non Detectable |
| HDS065 | 30/07/2019 | Positif | Positif | Non Detectable | Non Detectable |
| HDS053 | 26/07/2019 | Positif | Positif | Non Detectable | Non Detectable |
| HDS038 | 25/07/2019 | Positif | Positif | Non Detectable | Non Detectable |
| B-PTMEVHB014B | 14/06/2021 | Positif | Positif | Non Detectable | Non Detectable |
| B-PTMEVHB028B | 16/12/2021 | Positif | Positif | Non Detectable | Non Detectable |
| B-PTMEVHB023B | 11/05/2021 | Positif | Positif | Non Detectable | Non Detectable |
| B-PTMEVHB017B | 11/05/2021 | Positif | Positif | Non Detectable | Non Detectable |
| OBA046 | 29/07/2019 | Positif | Positif | Non Detectable | Non Detectable |
| B-PTMEVHB007B | 24/04/2021 | Positif | Positif | < 0,4 | < 15 |
| PTMEVHB573 | 17/06/2020 | Positif | Positif | < 0,4 | < 15 |
| PTMEVHB032 | 13/02/2019 | Positif | Positif | < 0,4 | < 15 |
| B-PTMEVHB013B | 12/05/2021 | Positif | Positif | < 0,4 | < 15 |
| PTMEVHB1124 | 15/12/2020 | Positif | Positif | 4.47 | 29817 |
| PTMEVHB348 | 21/08/2019 | Positif | Positif | 4.31 | 28182.1 |
| B-PTMEVHB024B | 12/05/2021 | Positif | Positif | 3.58 | 21450.82 |
| B-PTMEVHB015B | 21/06/2021 | Positif | Positif | 3.44 | 20813.72 |
| MFO025 | 25/07/2019 | Positif | Positif | 3.3 | 10521.41 |
| PTMEVHB565 | 11/03/2020 | Positif | Positif | 2.66 | 2535.15 |
| B-PTMEVHB005B | 19/04/2021 | Positif | Positif | 2.47 | 1654.12 |
| HDS044 | 25/07/2019 | Positif | Positif | 2.28 | 1077.03 |
| PTMEVHB882 | 17/09/2020 | Positif | Positif | 2.2 | 905.73 |
| OBA029 | 24/07/2019 | Positif | Positif | 2.095 | 697.8 |
| OBA031 | 24/07/2019 | Positif | Positif | 1.96 | 541.54 |
| B-PTMEVHB002B* | 05/04/2021 | Positif | Positif | 1.54 | 194.59 |
| B-PTMEVHB020B | 20/09/2021 | Positif | Positif | 1.34 | 125.04 |
| B-PTMEVHB012B | 10/05/2021 | Positif | Positif | 1.25 | 100.71 |
| PTMEVHB908 | 16/09/2020 | Positif | Positif | 0.45 | 15.82 |
| B-PTMEVIH/VHB03B | 07/02/2022 | Positif | Positif | Non Detectable | Non Detectable |
| B-PTMEVHB038B | 16/05/2022 | Positif | Positif | Non Detectable | Non Detectable |
| B-PTMEVHB036B | 21/03/2022 | Positif | Positif | Non Detectable | Non Detectable |
| B-PTMEVHB035B | 07/03/2022 | Positif | Positif | Non Detectable | Non Detectable |
| B-PTMEVHB034B | 21/01/2022 | Positif | Positif | Non Detectable | Non Detectable |
| B-PTMEVHB033B | 21/01/2022 | Positif | Positif | Non Detectable | Non Detectable |
| B-PTMEVHB040B | 26/06/2022 | Positif | Positif | Non Detectable | Non Detectable |
| B-PTMEVHB041B | 27/06/2022 | Positif | Positif | Non Detectable | Non Detectable |
| PTMEVHB121 | 22/03/2019 | Négatif | Positif |  |  |
| B-PTMEVHB/VIH 001B | 10/05/2021 | Négatif | Positif |  |  |
| B-PTMEVHB/VIH002B | 17/05/2021 | Négatif | Positif |  |  |
| PTMEVHB671 | 03/08/2020 | Négatif | Positif |  |  |
| PTMEVHB552 | 04/03/2020 | Négatif | Positif |  |  |
| PTMEVHB282 | 28/06/2019 | Négatif | Positif |  |  |
| MFO027 | 25/07/2019 | Négatif | Positif |  |  |
| MBY020 | 24/07/2019 | Négatif | Positif |  |  |
| B-PTMEVHB011B | 10/05/2021 | Négatif | Positif |  |  |
| B-PTMEVHB010B | 03/05/2021 | Négatif | Positif |  |  |
| B-PTMEVHB009B | 03/05/2021 | Négatif | Positif |  |  |
| B-PTMEVHB006B | 24/04/2021 | Négatif | Positif |  |  |
| B-PTMEVHB004B | 19/04/2021 | Négatif | Positif |  |  |
| B-PTMEVHB003B | 12/04/2021 | Négatif | Positif |  |  |
| PTMEVHB553 | 04/03/2020 | Négatif | Positif |  |  |
| PTMEVHB366 | 09/09/2019 | Négatif | Positif |  |  |
| MBY037 | 29/07/2019 | Négatif | Positif |  |  |
| B-PTMEVHB001B/B-PTMEVHB008B | 05/04/2021 | Négatif | Positif |  |  |
| PTMEVHB1009 | 19/10/2020 | Négatif | Positif |  |  |
| PTMEVHB686 | 06/04/2020 | Négatif | Positif |  |  |
| PTMEVHB521 | 12/02/2020 | Négatif | Positif |  |  |
| PTMEVHB916 | 22/09/2020 | Négatif | Positif |  |  |
| PTMEVHB1173 | 17/03/2021 | Négatif | Positif |  |  |
| PTMEVHB962 | 05/10/2020 | Négatif | Positif |  |  |
| HDS060 | 30/07/2019 | Négatif | Positif |  |  |
| PTMEVHB976 | 07/10/2020 | Négatif | Positif |  |  |
| PTMEVHB952 | 24/09/2020 | Négatif | Positif |  |  |
| PTMEVHB949 | 23/09/2020 | Négatif | Positif |  |  |
| PTMEVHB914 | 21/09/2020 | Négatif | Positif |  |  |
| PTMEVHB904 | 19/09/2020 | Négatif | Positif |  |  |
| PTMEVHB890 | 18/09/2020 | Négatif | Positif |  |  |
| PTMEVHB871 | 16/09/2020 | Négatif | Positif |  |  |
| PTMEVHB866 | 08/09/2020 | Négatif | Positif |  |  |
| PTMEVHB847 | 02/09/2020 | Négatif | Positif |  |  |
| PTMEVHB836 | 02/09/2020 | Négatif | Positif |  |  |
| PTMEVHB821 | 01/09/2020 | Négatif | Positif |  |  |
| PTMEVHB804 | 27/08/2020 | Négatif | Positif |  |  |
| PTMEVHB771 | 25/08/2020 | Négatif | Positif |  |  |
| PTMEVHB709 | 10/08/2020 | Négatif | Positif |  |  |
| PTMEVHB525 | 19/02/2020 | Négatif | Positif |  |  |
| PTMEVHB478 | 05/12/2019 | Négatif | Positif |  |  |
| PTMEVHB443 | 06/11/2019 | Négatif | Positif |  |  |
| PTMEVHB441 | 06/11/2019 | Négatif | Positif |  |  |
| PTMEVHB399 | 07/10/2019 | Négatif | Positif |  |  |
| PTMEVHB310 | 16/07/2019 | Négatif | Positif |  |  |
| PTMEVHB307 | 16/07/2019 | Négatif | Positif |  |  |
| PTMEVHB270 | 20/06/2019 | Négatif | Positif |  |  |
| PTMEVHB221 | 27/05/2019 | Négatif | Positif |  |  |
| PTMEVHB187 | 30/04/2019 | Négatif | Positif |  |  |
| PTMEVHB186 | 29/04/2019 | Négatif | Positif |  |  |
| PTMEVHB156 | 11/01/1900 | Négatif | Positif |  |  |
| PTMEVHB143 | 08/04/2019 | Négatif | Positif |  |  |
| PTMEVHB1168 | 25/01/2021 | Négatif | Positif |  |  |
| PTMEVHB1163 | 19/01/2021 | Négatif | Positif |  |  |
| PTMEVHB1159 | 19/01/2021 | Négatif | Positif |  |  |
| PTMEVHB1119 | 10/12/2020 | Négatif | Positif |  |  |
| PTMEVHB1114 | 08/12/2020 | Négatif | Positif |  |  |
| PTMEVHB1107 | 08/12/2020 | Négatif | Positif |  |  |
| PTMEVHB1096 | 07/12/2020 | Négatif | Positif |  |  |
| PTMEVHB105 | 15/03/2019 | Négatif | Positif |  |  |
| PTMEVHB1015 | 19/10/2020 | Négatif | Positif |  |  |
| PTMEVHB1012 | 19/10/2020 | Négatif | Positif |  |  |
| PTMEVHB101 | 14/03/2019 | Négatif | Positif |  |  |
| PTMEVHB051 | 20/02/2019 | Négatif | Positif |  |  |
| PTMEVHB012 | 04/02/2019 | Négatif | Positif |  |  |
| OBA021 | 24/07/2019 | Négatif | Positif |  |  |
| OBA013 | 24/07/2019 | Négatif | Positif |  |  |
| MFO057 | 31/07/2019 | Négatif | Positif |  |  |
| MBY060 | 30/07/2019 | Négatif | Positif |  |  |
| MBY042 | 29/07/2019 | Négatif | Positif |  |  |
| MBY031 | 25/07/2019 | Négatif | Positif |  |  |
| HDS055 | 29/07/2019 | Négatif | Positif |  |  |
| HDS015 | 24/07/2019 | Négatif | Positif |  |  |
| B-PTMEVHB027B | 13/12/2021 | Négatif | Positif |  |  |
| B-PTMEVHB026B | 14/05/2021 | Négatif | Positif |  |  |
| B-PTMEVHB025B | 13/05/2021 | Négatif | Positif |  |  |
| B-PTMEVHB022B | 18/10/2021 | Négatif | Positif |  |  |
| B-PTMEVHB021B | 27/09/2021 | Négatif | Positif |  |  |
| B-PTMEVHB019B | 06/09/2021 | Négatif | Positif |  |  |
| B-PTMEVHB018B | 26/07/2021 | Négatif | Positif |  |  |
| B-PTMEVHB016B | 12/07/2021 | Négatif | Positif |  |  |
| B-PTMEVHB039B | 23/05/2022 | Négatif | Positif |  |  |
| B-PTMEVHB037B | 28/04/2022 | Négatif | Positif |  |  |
| B-PTMEVHB032B | 24/01/2022 | Négatif | Positif |  |  |
| B-PTMEVHB031B | 24/01/2022 | Négatif | Positif |  |  |
| B-PTMEVHB030B | 17/01/2022 | Négatif | Positif |  |  |
| B-PTMEVHB029B | 17/01/2022 | Négatif | Positif |  |  |
| B-PTMEVHB042B | 04/07/2022 | Négatif | Positif |  |  |
| B-PTMEVHB043B | 05/09/2022 |  | Positif |  |  |
| B-PTMEVHB044B | 12/09/2022 |  | Positif |  |  |
| B-PTMEVHB045B | 03/10/2022 |  | Positif |  |  |
|  |  |  |  |  |  |
| HDV: hepatitis delta virus | | HBsAg: hepatitis B surface antigen | | |  |
